# Metagenomic Approach with the NetoVIR Enrichment Protocol Reveals Virus Diversity within Ethiopian Honey Bees (*Apis mellifera simensis*)

**DOI:** 10.3390/v12111218

**Published:** 2020-10-27

**Authors:** Haftom Gebremedhn, Ward Deboutte, Karel Schoonvaere, Peter Demaeght, Lina De Smet, Bezabeh Amssalu, Jelle Matthijnssens, Dirk C. de Graaf

**Affiliations:** 1 Laboratory of Molecular Entomology and Bee Pathology, Ghent University, B-9000 Ghent, Belgium; haftush@yahoo.com (H.G.); karel.schoonvaere@ugent.be (K.S.); peter.demaeght@ugent.be (P.D.); lina.desmet@ugent.be (L.D.S.); 2 Tigray Agricultural Research Institute, P.O. Box 492 Mekelle, Ethiopia; 3 Laboratory of Viral Metagenomics, Laboratory for Clinical and Epidemiological Virology, Rega Institute, Department of Microbiology, Immunology and Transplantation, University of Leuven, B-3000 Leuven, Belgium; ward.deboutte@kuleuven.be (W.D.); jelle.matthijnssens@kuleuven.be (J.M.); 4 Holeta Bee Research Center, P.O. Box 22 Holeta, Ethiopia; amssalub@gmail.com

**Keywords:** honey bee (*Apis mellifera*), bee viruses, *Varroa destructor*, metagenomics, virome, DNA viruses, RNA viruses, plant-specific, insect-specific, virus tolerance

## Abstract

Metagenomics studies have accelerated the discovery of novel or divergent viruses of the honey bee. However, most of these studies predominantly focused on RNA viruses, and many suffer from the relatively low abundance of viral nucleic acids in the samples (i.e., compared to that of the host). Here, we explored the virome of the Ethiopian honey bee, *Apis mellifera simensis*, using an unbiased metagenomic approach in which the next-generation sequencing step was preceded by an enrichment protocol for viral particles. Our study revealed five well-known bee viruses and 25 atypical virus species, most of which have never been found in *A. mellifera* before. The viruses belong to *Iflaviridae*, *Dicistroviridae*, *Secoviridae*, *Partitiviridae*, *Parvoviridae*, *Potyviridae,* and taxonomically unclassified families. Fifteen of these atypical viruses were most likely plant-specific, and the remaining ten were presumed to be insect-specific. Apis mellifera filamentous virus (AmFV) was found in one sampling site out of 10. Two samples contained high read counts of a virus similar to Diatraea saccharales densovirus (DsDNV), which is a virus that causes high mortality in the sugarcane borer. AmFV and the DsDNV-like virus were the only DNA viruses found. Three viruses that primarily infect *Drosophila* spp. were also discovered: La Jolla virus (LJV), Kilifi virus (KiV), and Thika virus. Our study suggests that phoretic varroa mites are involved in the transmission of LJV and KiV and that both viruses replicate in mites and adult bees. We also found an overwhelming dominance of the deformed wing virus type B variant, which fits well with the apparently harmless infestation by *Varroa destructor*. It was suggested that Ethiopian bees have developed tolerance against virus infections as the result of natural selection.

## 1. Introduction

Honey bees play a significant role in boosting crop production through pollination [1,2]. It is estimated that one-third of the world’s food crop production relies on pollination largely by bees [3]. Worldwide, the total economic value of insect pollination reached €153 billion in 2005 [1]. Despite the importance of honey bees as a pollinator, the beekeeping industry is facing high honey bee colony losses [4,5,6,7], which may in the long run threaten food production.

Globally, viral pathogens and *Varroa destructor* mites are considered the most serious biotic threats to honey bees’ health [5,8,9,10]. The mite affects honey bee health in many ways, for instance by direct feeding on the bees’ fat body tissue [11], by weakening their immune system [12], by acting as a vector of viral pathogens [13,14,15] and by activating the replication of these viruses [16], leading to an amplification of viral loads in individual bees and of the colony as a whole [17]. Moreover, the mite acts as a viral reservoir, as it can carry high virus loads by feeding or active virus replication [15,18]. The clinical signs and consequences of viral diseases of honey bees are diverse and include wing deformities, loss of hair, paralysis, trembling, and complications during larval growth, which eventually can lead to a shorter lifespan or death [19,20,21]. One virus in particular, the deformed wing virus (DWV), was demonstrated to have a mutualistic symbiotic relationship with the varroa mite [12], to such an extent that the clinical signs of the virus were for a long time wrongly attributed to the mite [13]. Moreover, several studies indicate that virus infections can be considered an important stressor of honey bees and contribute to the colony losses [22,23].

In Africa, the presence of common honey bee viruses such as DWV, Lake Sinai virus (LSV), acute bee paralyse virus (ABPV), Israeli acute paralysis virus (IAPV), chronic bee paralysis virus (CBPV), sacbrood virus (SBV), black queen cell virus (BQCV), and Apis mellifera filamentous virus (AmFV) have been confirmed in different parts of the continent, including Kenya, Uganda, and Sudan [24,25,26,27,28,29,30]. However, most of these virus studies conducted in Africa performed virus-specific RT-PCR tests to monitor the presence of previously known honey bee viruses, which does not provide insights into the so far undiscovered viruses that circulate in bees or other viruses to which bees might be exposed. However, there are reasons to believe that the virus diversity of bees in Africa might differ from those in other parts of the world. Indeed, recent high-throughput sequencing studies in geographically distinct honey bee populations identified several novel viruses, some of which were region-specific [31,32]. Even in varroa-free Australian honey bees, a diverse *Picornavirales* virome could be discovered [33]. Furthermore, the spill-over of bee pathogens—including RNA viruses—from managed honey bees to wild bees is well documented [34,35]. Pathogens and mutualistic microbes alike may be transferred horizontally between species by feeding on the same food source (e.g., nectar) [36]. Given the unique diversity of bee pollinator communities in some geographic regions of the world, including Africa [37], it seems reasonable to believe that the set of viruses to which honey bees are exposed may differ also.

The advent of whole-community genome sequencing or metagenomics opened a new opportunity to understand the genetic diversity of the viral community without a priori knowledge of the virus type present in a sample [38]. It hugely increased our knowledge of the virus world [39] and has accelerated the discovery of novel or divergent viruses in the honey bee, *Apis mellifera* [31,32,40]. However, most of the viral metagenome studies on honey bees predominantly focused on RNA viruses [32,41,42]. Moreover, despite the relatively low abundance of viral nucleic acids in the samples (i.e., compared to that of the host), many viral metagenomics studies in bees were performed without a proper enrichment protocol prior to sequencing of the viral genomes [32,41], although such an enrichment step represents a critical factor that largely affects the discovery success of the metagenomics study [43,44]. Therefore, it is arguable that the diversity of viruses associated with bees is not yet fully understood.

This study was designed to explore the diversity of viruses in Ethiopian honey bees (*Apis mellifera simensis*) using an unbiased metagenomics approach. For the viral metagenomics analysis, we have employed the NetoVIR protocol: a reproducible protocol that enriches virus-like particles by removing non-viral nucleic acids, enabling the detection of both RNA and DNA viruses [45]. This approach revealed five well-known honey bee viruses and 25 other viruses, most of which were never found in *A. mellifera* before. Two of these atypical viruses were further investigated, aiming to show that they actually infect honey bees and how the transmission might occur.

## 2. Materials and Methods

### 2.1. Study Area

**Figure 1:**
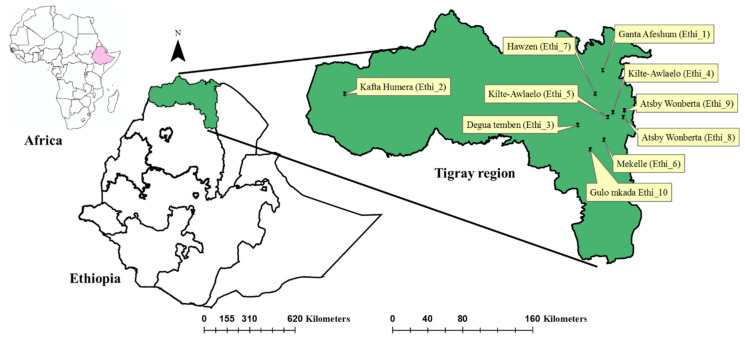
Map of Ethiopia in the horn of East Africa (see left window; Ethiopia is colored pink). The selected apiary sites in the Tigray National Regional State of Ethiopia (in green) are marked, and their sample codes are given (Ethi_1 to Ethi_10).

The study was conducted in Ethiopia, which is found in the horn of East Africa. The study sites included eight representative districts (Ganta Afeshum, Kilte Awlaelo, Gulo Mkada, Degua Tembien, Hawzen, Kafta Humera, Mekelle, and Atsby Wonberta) from the Tigray National Regional State of Ethiopia (Figure 1). Access to transport, honey production potential, agro-ecology, and distance to the laboratory of Tigray Agricultural Research Institute were considered to select the districts.

### 2.2. Sample Collection and Transportation

Samples were collected (with the owners’ permission) between August and October 2017 (i.e., in the active season) from 20 bee colonies spread over 10 apiaries (two colonies each; Figure 1). Three types of samples were collected: adult bees, black-headed pupae, and varroa mites. Approximately 50 guard bees [46] were collected late at night at the hive entrance using a torch light. Capped worker bee brood cells were opened to collect black-headed pupae and the parasitizing mites, if present. Samples were stored at −24 °C at the Laboratory of Biotechnology, Mekelle Agricultural Research Center, before shipping to Ghent University while keeping them frozen (on dry ice), where they were stored at −80 °C until further testing. The laboratory analyses were carried out both at the Laboratory of Viral Metagenomics of the Rega Institute of KU Leuven and the Laboratory of Molecular Entomology and Bee Pathology of Ghent University.

### 2.3. Homogenization, Centrifugation and Filtration

Samples derived from adult bees were used for metagenomics analysis. Two bees per hive were homogenized separately using a tissue homogenizer (Minilys, Bertin GMBH, Frankfurt am Main, Germany) at 3000 rpm for 1 min in the presence of 500 μL sterile phosphate-buffered saline (PBS) and three zirconium beads. After centrifugation at 17,000× *g* for 3 min, supernatants of two individual samples obtained from the same apiary were pooled, filtered through a 0.8 μm filter (PES, polyethersulfone), and centrifuged at 17,000× *g* for 1 min.

### 2.4. Nuclease Treatment and Nucleic Acid Extraction

After filtration, samples were subjected to nuclease treatment to remove non-viral nucleic acids including host contaminants (while viral genetic material remains protected by their capsid). Briefly, 130 μL of the filtrate was mixed with 2 μL of benzonase nuclease, 1 μL of micrococcal nuclease and 7 μL of 20× homemade buffer (1 M Tris, 100 mM CaCl_2_ and 30 mM MgCl_2_, pH 8) and incubated at 37 °C for 2 h. Then, 7 μL of 10 nM EDTA was added to inactivate the enzymes. The total nucleic acids (i.e., RNA and DNA) were extracted using the QIAamp Viral RNA Mini preparation kits (Qiagen, Hilden, Germany) according to the manufacturer’s instructions, without using carrier RNA.

### 2.5. cDNA Synthesis, Amplification and Purification

cDNA synthesis and PCR amplification was performed according to the modified version of the Whole Transcriptome Amplification (WTA2, SigmaAldrich, St. Louis, MO, USA) kit [45]. Amplicons were purified using the MSB^®^ SPIN PCRAPACE purification kit (Stratec Biomedical, Birkenfeld, Germany), according to manufacturer’s protocol. The concentration of the purified DNA was quantified using the Qubit dsDNA HS assay kit, according to the manufacturer’s protocol and then diluted to 1.2 ng/µL.

DNA libraries were prepared using the Nextera XT DNA library preparation kit (Illumina, San Diego, CA, USA) as per the manufacturer’s instructions, and libraries were purified following the Agencourt^®^ AMPure^®^ PCR purification protocol. The concentration of the purified library was quantified using a Qubit dsDNA HS assay kit, according to the manufacturer’s instructions. Then, the DNA library size distribution and quality were evaluated using an hsDNA chip on a Bioanalyzer platform (Agilent Technologies, 2009, Santa Clare, CA, USA). Sequencing was performed using the Illumina NextSeq platform for paired-end sequencing with a base length of 2 × 150 bp, and an average of 9,304,652 reads were retrieved per sample.

### 2.6. Data Processing

The bioinformatics workflow is illustrated in Figure S1. The quality of raw reads obtained from Illumina NextSeq was evaluated using FastQC (https://www.bioinformatics.babraham.ac.uk/projects/fastqc/). After quality assessment, sequencing adapters and low-quality sequences with Phred scores below 20 were trimmed using Trimmomatic v0.38 [47]. Trimmed reads were assembled per library using metaSPAdes assembler v: 3.11.1 [48] with kmers = 21, 33, 55, and 77. Subsequently, scaffolds were collapsed at 95% nucleotide sequence identity over 80% of the length of the longest scaffold (https://bitbucket.org/MAVERICLab/docker-clustergenomes). The resulting set of non-redundant scaffolds were compared to the NCBI non-redundant protein database (downloaded on 22 January 2018) using DIAMOND BLASTx [49] (E-value < 1 × 10^−3^). Sequences that showed no homology to any sequences present in the non-redundant protein database were extracted and compared to the NCBI non-redundant nucleotide database (downloaded on 22 January 2018) using tBLASTx (E-value < 1 × 10^−5^). BLAST output was visualized and taxonomies were assigned using the KronaTools software [50]. The abundance of each taxon was estimated by mapping quality trimmed reads against a sequence set containing the longest non-redundant scaffold using BWA v 0.7.16 [51]. Only sequences longer than 200 bp represented by at least 1000 reads were considered genuine.

### 2.7. Data Accessibility

Sequencing data have been deposited in the SRA database (NCBI) under BioProject accession number PRJNA559070 and BioSample accession number SAMN12512743- SAMN12512752. La Jolla virus (LJV), Kilifi virus (KiV), and Thika virus (ThV) near-complete genome sequences were deposited in GenBank under accession numbers MT681680, MT681681, and MT681679, respectively. A multifasta file of the sequences retrieved in this study and used in the phylogenies of the known honey bee viruses is provided as a Supplementary File.

### 2.8. Virus Quantification and Detection Using RT-qPCR

Quantification of the virus loads of adult honey bees in each honey bee colony under investigation was performed using RT-PCR for a selected set of five viruses: DWV, BQCV, SBV, LJV, and KiV. Later on, these analyses were extended with the detection of LJV and KiV in varroa mites and honey bee pupae (i.e., infested and not infested by varroa mites) collected from the same colonies.

Ten adult bees per colony (*n* = 20) or ten pupae per apiary (i.e., 5 bees/colony) were homogenized using a Bullet Blender Storm 5^®^/VISUM IDPBW instrument (at maximum speed) in 5 mL phosphate-buffered saline (PBS), 0.25 mL zirconia beads, and three metal beads. The homogenate (1 mL) was centrifuged at 1500 rpm for 10 min. Then, the supernatant was transferred to a new tube and centrifuged at maximum speed for 15 min. For mites, 10 mites per apiary (i.e., 5 mites/colony) were crushed with a plastic pestle in a 1.5 mL Eppendorf tube containing 500 µL of Qiazol lysis reagent. After homogenization of the mite sample, 500 µL Qiazol lysis reagent was added, and the sample was incubated for 5 min. Next, 200 µL of chloroform was added, incubated for 2–3 min, and centrifuged at 12,000× *g* for 15 min. We used the RNeasy Lipid Tissue mini kit (Qiagen, Hilden, Germany) to extract RNA from adult bees following the manufacturers’ instructions. RNA from pupa and mite samples was extracted using the QIAamp^®^ viral RNA kit (Qiagen, Hilden, Germany) and RNeasy Lipid Tissue mini kit (Qiagen, Hilden, Germany), respectively. The cDNA was synthesized from 5 µL RNA template using the RevertAid H Minus First Strand cDNA Synthesis kit (Thermo Fisher Scientific, Waltham, MA, USA) with random hexamer primers, according to the manufacturer’s instructions.

For the RT-qPCR assays, the Platinum^®^ SYBR^®^ Green qPCR SuperMix-UDG kit (Thermo Fisher Scientific, Waltham, MA, USA) was used. Each reaction consisted of 12.5 µL of Platinum^®^ SYBR^®^ Green qPCR SuperMix-UDG, 0.5 µL forward primer (10 µm), 0.5 µL reverse primer (10 µm), 10.5 µL DEPC-treated water, and 1 µL of cDNA template. To quantify the viral load in adult bees, we used 2 µL of cDNA template and 9.5 µL of DEPC-treated water, while the amount of the other reagents remained the same. The RT-qPCR reaction was carried out on the CFX96 Real-Time PCR Detection System (Bio-Rad, Hercules, CA, USA) using the following program: 50 °C for 2 min, 95 °C for 2 min, and 40 cycles of a combined denaturation (15 sec at 95 °C) and annealing (30 sec at 60 °C) step. At the end of this program, a melt curve was generated by measuring fluorescence after each temperature increase of 0.5 °C for 5 sec over a range from 65 to 95 °C to verify the presence of the desired amplicon. PCR reactions to quantify viruses in adult bees was performed in triplicate, whereas the virus detection in mites and pupae was performed in duplicate. No template controls (water) were included in each assay. Primers for the detection of KiV and LJV were designed directly from the NGS sequencing data using Primer3plus software https://www.ncbi.nlm.nih.gov/tools/primer-blast/. All primers used are given in Table S1 [52,53].

To enable the absolute quantification of LJV and KiV, plasmid standards containing the viral of LJV and KiV were constructed using a TOPO TA Cloning Kit for sequencing, according to the manufacturer’s instructions. Then, the plasmids were isolated with the GeneJet plasmid Miniprep kit (Thermo Fisher Scientific, Waltham, MA, USA), according to the manufacturer’s instructions. The viral copy number per bee was obtained by multiplying the reported qPCR copy number values by a correction factor 300× copies/PCR. This conversion factor was calculated based on the volume used at each step of the RT-qPCR method [54].

### 2.9. Phylogenetic Analysis

Retrieved, near-complete (redundant) genome sequences of DWV, BQCV, LSV, SBV, LJV, KiV, and ThV were used for phylogenetic analysis. Relevant reference sequences, retrieved from NCBI GenBank and ENA using BLASTn, were also included. For LJV, KiV, and ThV, BLASTp hits were used. Multiple sequence alignments were made on either nucleotide level (DWV, BQCV, SBV, and LSV) or on an amino acid level (LJV, KiV, ThV, BQCV, and SBV) using MAFFT [55] on the L-INS-i setting. Resulting alignments were trimmed using trimAl [56], with the gappyout setting. Phylogenetic trees were subsequently constructed with IQ-TREE [57], using automated model selection [58] and bootstrapped using 1000 ultrafast bootstraps [59]. Resulting trees were midpoint-rooted (or outgroup rooted, for the amino acid trees of BQCV and SBV) using the phangorn package [60] and visualized using the ggtree package [61], implemented in R version 3.6.2 (https://www.R-project.org).

### 2.10. Negative Strand Detection Using RT-MLPA

Multiplex Ligation Probe Amplification (MLPA) probes and Reverse Transcriptase (RT) primers were designed for KiV and LJV negative strands. The MLPA probes were designed as described previously [62]. Custom RT primers were designed using primer 3 (http://bioinfo.ut.ee/primer3/). They were chosen immediately adjacent to the MLPA probes, with no more than 15 nucleotides between the last nucleotide and the first nucleotide of the probe sequence. Negative strands were synthetically designed and used as a template for the positive control. All primers, probes, and synthetic templates were synthesized by Integrated DNA Technologies (Leuven, Belgium) and are listed in Table S1.

RT-MLPA analysis was performed on pooled samples (10 bees or mites per sample, see Section 2.8) as described by De Smet et al. [62]. Briefly, 1 µL of RNA was reverse transcribed using custom-made RT primers and 30U MMLV reverse transcriptase (Promega, Madison, WI, USA). The 6 µL reaction contained 0.5 µL RT primer/dNTP mix (5 pmol/µL RT primer, 5 mM dNTPs) and was incubated for 1 min at 80 °C, followed by 5 min at 45 °C. Next, the reverse transcriptase was added, and the reaction was incubated for 15 min at 37 °C, followed by an inactivation for 2 min at 98 °C. A probe mix containing the half-probe oligo’s for negative strand detection was prepared for each virus and contained 1.33 fmol/µL of each oligo. A mixture of 1.5 µL of probe mix and 1.5 µL of MLPA buffer was added to each RT reaction and hybridized overnight at 60 °C, after an initial denaturation step of 1 min at 95 °C. After hybridization, the probes were ligated together using the Ligase-65 enzyme for 15 min at 54 °C, followed by inactivation of the ligase for 5 min at 98 °C. Finally, a mixture of 2 µL of SALSA primer mix and 0.5 µL of SALSA polymerase was added to each reaction, and amplification was performed for 35 cycles (30 sec at 95 °C, 30 sec at 58 °C, and 1 min at 72 °C) with a final extension step for 20 min at 72 °C. All MLPA reagents were obtained from MRC Holland (Amsterdam, the Netherlands). Analysis of the MLPA products was performed using 4% high-resolution agarose gel electrophoresis.

## 3. Results

### 3.1. High-Level Taxonomic Classification

**Figure 2:**
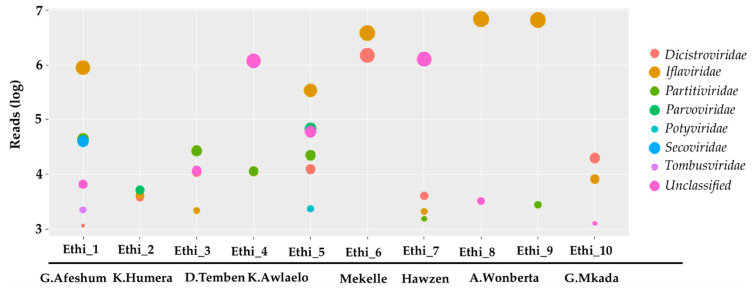
Identified viral families and the numbers of reads assigned to each family. Circle radii indicate the number of reads (log10 transformed) assigned to each family per sample. Ethi_1 to Ethi_10 refers to the sample code given to each apiary, and district names are given below (see Figure 1).

Illumina sequencing generated a total of 93.05 million paired-end sequence reads of 150 bp in length (PRJNA559070). Around 63.5% (59.1 million reads) of the reads belonged to scaffolds that shared sequence homology with known taxa in the non-redundant protein and nucleotide databases (NCBI). The majority of the reads could be assigned to Eukaryota taxa (28.5 million reads) followed by virus (25.6 million reads) and bacteria (4.2 million reads). The viral reads mapped to members of several eukaryotic virus families including *Dicistroviridae*, *Iflaviridae*, *Partitiviridae*, *Parvoviridae*, *Potyviridae*, *Secoviridae*, *Tombusviridae*, and other taxonomically unclassified families (Figure 2). The majority of the viral sequences detected in our samples could be assigned to the family *Iflaviridae* (20.25 million reads).

Our viral metagenomics analysis revealed both DNA and RNA viruses (Tables S2–S4), although most of the viral taxa assigned have an RNA genome (93.3%). This finding was also reflected by the absolute number of viral reads. The total number of reads assigned to each virus across the different localities is summarized in Tables S2–S4.

### 3.2. Presence of Known Honey Bee Viruses

**Figure 3:**
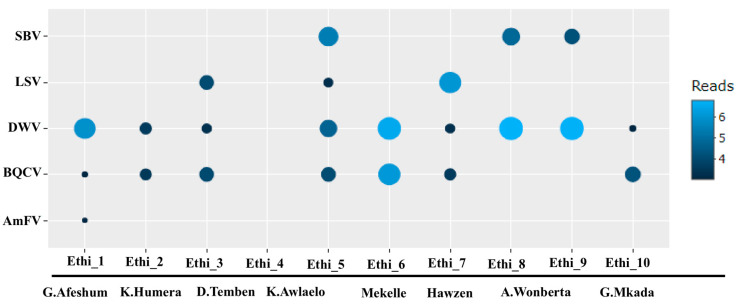
Identified known honey bee viruses and numbers of reads assigned based on the sequencing data. Circle radii and colors indicate the number of reads (log10 transformed) assigned to each known honey bee virus per sample. Ethi_1 to Ethi_10 refers to the sample code given to each apiary, and district names are given underneath (see Figure 1).

Our study identified five known honey bee viruses: DWV, SBV, BQCV, AmFV, and LSV (Table S2; Figure 3) in honey bee samples. The most prevalent viruses among all sampling sites were DWV and BQCV, each of which was detected in nine and seven out of 10 sampling sites, respectively. Sequences related to SBV and LSV were present in three out of 10 sampling sites. The dsDNA virus AmFV was found in one sampling site out of 10. Further, the DWV, BQCV, and SBV retrieved in this study were represented by consensus sequences of 10986-nt, 8504-nt, and 8770-nt long, and covered 92%, 99%, and 97% of the complete reference genomes deposited in GenBank, respectively. Roughly 83.9.3% (21.5 million reads) of the viral reads belonged to known honey bee viruses. The vast majority of those could be assigned to DWV (18,309,545 reads), followed by BQCV (1,554,072 reads) (Figure 3).

### 3.3. Atypical Viruses

We identified 25 viruses, uncommon to honey bees, of which 15 showed homology to plant-specific viruses (Table S3) and 10 showed homology to insect-specific viruses (Table S4). Overall, the similarity of these atypical viruses to known bee viruses was rather low; they were present at a low level, representing only 0.62% (160,163 reads) and 5.3% (1,354,843 reads) of the viral reads, respectively. The distribution of these viruses was also low. Plant-specific viruses were present in one to five localities, and insect-specific viruses were present in one to two localities.

The plant-specific viruses belong to the families *Partitiviridae* (n = 10), *Secoviridae* (n = 3), *Tombusviridae* (n = 1), and *Potyviridae* (n = 1) (Table S3). The identified viruses that seem insect-specific are related to known viruses that possess RNA and DNA genomes. These included LJV (*Iflaviridae* family), KiV (unclassified *Picornavirales*), Diatraea saccharalis densovirus (DsDNV) (*Parvoviridae* family), and eight unclassified/unassigned viruses (Thika virus (ThV), Wenzhou sobemo-like virus 4, Hubei mosquito virus 2, Hubei toti-like virus 2, Hubei partiti-like virus 51, Hubei partiti-like virus 34, Hubei mosquito virus 2, and Wuhan insect virus 27). LJV, KiV, and ThV were found in two, two, and one out of 10 sampling sites, respectively (Table S4), and their scaffolds were 10,262-nt, 8963-nt, and 6169-nt long, respectively. They showed 96.6%, 85.2%, and 97.3% amino acid identity with LJV (GenBank: YP_009140562), KiV (GenBank: AYQ66683), and ThV (GenBank:YP_009140561.1) isolated from the *Drosophila melanogaster* in Ghana, USA and the United Kingdom, respectively [63]. The found that Wenzhou sobemo-like virus 4 showed 75.26% amino acid identity with the Guadeloupe mosquito virus sequences (QEM39285.1) obtained from mosquitos in the French island of Guadeloupe in the Caribbean. The ssDNA virus DsDNV was found in two samples (Ethi_2 and Ethi_5) with very high read counts (74,733 reads) and shared 46.1% identity with Diatraea saccharalis densovirus sequences isolated from *Diatraea saccharalis* (NP_046815.1) and will be referred to as a DsDNV-like virus. Partitiviruses are dsRNA viruses and have been observed mainly in plants and fungi, but recently, they were also described as part of the bee virosphere [31]. The Hubai partiti-like virus 51 was found in two samples (Ethi_5 and Ethi_5) with almost 3 million reads and shows 77% amino acid identity with the RNA-dependent RNA polymerase of Hubai partiti-like virus 51 (GenBank: APG78321.1).

### 3.4. Virus Quantification

We quantified the virus loads of adult bees from all colonies under investigation for a selected set of five viruses. DWV, BQCV, and SBV were chosen because they were commonly found in bees in Africa [24,26,27,30] and beyond [10]. LJV has recently been discovered in honey bees in Australia [33], but has so far not been found elsewhere. KiV was chosen because its discovery seemed the most promising, given the fact that it was previously described in wild *Drosophila* in Africa [63].

**Figure 4:**
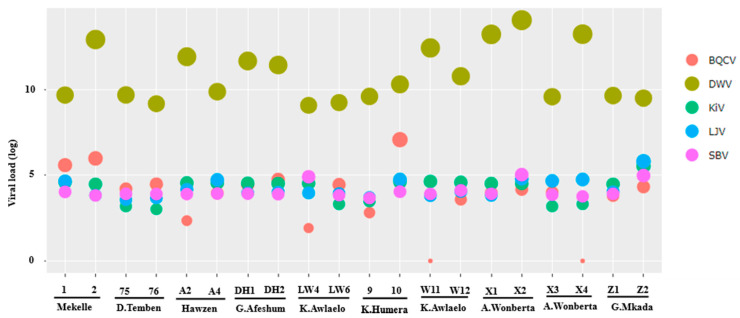
The load of black queen cell virus (BQCV), deformed wing virus (DWV), sacbrood virus (SBV), La Jolla virus (LJV) and Kilifi virus (KiV) per honey bee determined by qPCR. Circle radii indicates the number of virus copy number (log10 + 1 transformed) per bee (*Y*-axis). The indication 1 to Z2 refers to the code given to each tested honey bee colony, and district names are given underneath (see Figure 1).

The virus loads of the five selected viruses varied among the 20 tested honey bee colonies (Figure 4). DWV had the highest average viral load (7.6 × 10^12^ virus copies per bee) followed by BQCV (6.1 × 10^5^ virus copies per bee). Virus loads of SBV (2.1 × 10^4^ virus copies per bee), LJV (5.3 × 10^4^ virus copies per bee), and KiV (3.9 × 10^4^ virus copies per bee) were the lowest.

### 3.5. Phylogenetic Analysis

**Figure 5:**
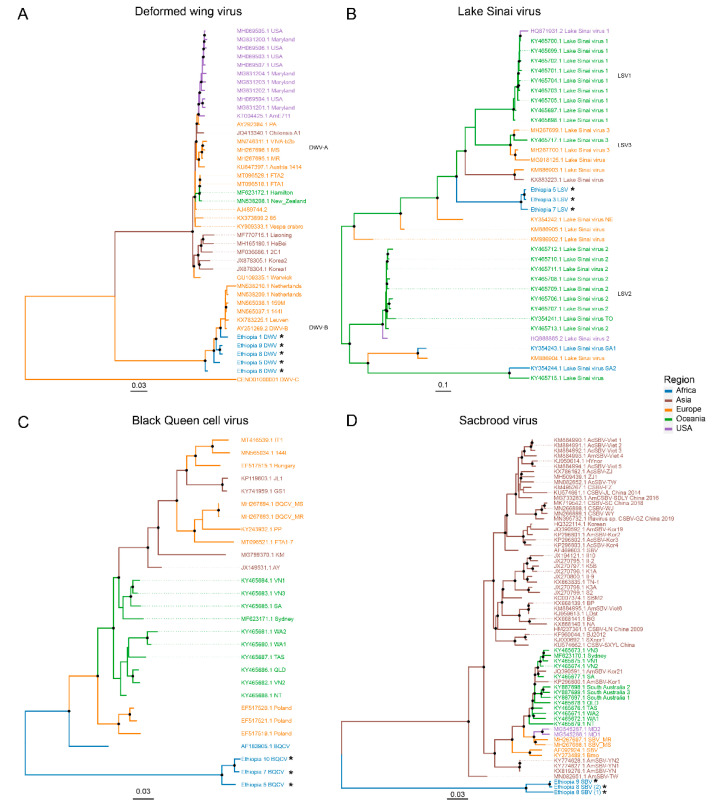
Phylogenies of known honey bee viruses: deformed wing virus (**A**), Lake Sinai virus (**B**), black queen cell virus (**C**) and sacbrood virus (**D**). Midpoint-rooted phylogenetic trees were drawn based on near-complete genome alignments (on nucleotide level). Bootstrap values exceeding 70% are indicated as black circles on their respective nodes. Tip labels as well as branch groups are colored according to the geographical location of which the sequence was derived. Sequences retrieved in this study are indicated in blue, with asterisks. A multifasta-file of these sequences is provided as Supplementary File.

Phylogenetic trees were constructed for DWV, BQCV, LSV, and SBV using the redundant, near-complete sequences retrieved in this study combined with their nearest BLASTn hits. DWV sequences obtained in this study showed an average nucleotide similarity of 97% with their respective GenBank hits. Retrieved sequences fell into the clade together with European DWV-B sequences. The Ethiopian strains were clustered relatively distant from established DWV-B strains, despite the overall high sequence similarity. The three retrieved LSV sequences were more divergent, showing an average nucleotide similarity of 75% with their respective GenBank hits. Phylogenetic analysis revealed the Ethiopian LSV strains paraphyletic to LSV-1 and LSV-2 species (Figure 5B). Retrieved BQCV sequences revealed an average nucleotide similarity of 83% to BQCV isolated in South Africa (AF183905.1). Phylogenetic analysis revealed that these sequences form an outgroup to all known near-complete BQCV sequences encompassed in GenBank (Figure 5C). A similar pattern could be observed for SBV, where the Ethiopian strains showed an average nucleotide similarity of 80% with their respective GenBank hits. The retrieved SBV sequences were placed as an outgroup respective to known SBV sequences (Figure 5D). Since both the retrieved SBV and BQCV sequences were placed as outgroups respective to known strains, amino acid level phylogenies were drawn using either Renmark bee virus 1 (MG995710.1) as an outgroup (for BQCV) or Darwin bee virus 4 (MG995699.1) (Figure S2). Phylogenetic placement of SBV in the amino acid tree was very similar to the phylogenetic placement in the nucleotide tree. The Ethiopian BQCV sequences fell in a sister clade with respect to another clade that mainly contained Australian sequences. However, none of the higher level clades had bootstrap support higher than 70%.

**Figure 6:**
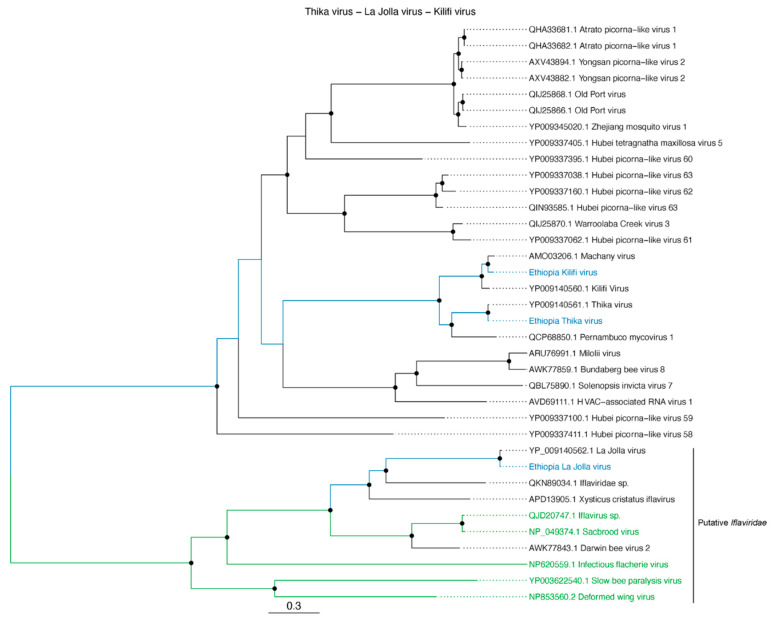
Midpoint-rooted phylogenetic tree based on near-complete genome alignments (on amino acid level) of the retrieved Ethiopian strains of KiV, Thika virus (ThV), and LJV. The putative *Iflaviridae* clade is highlighted. Assigned *Iflaviridae* members are indicated in green. Retrieved viruses in this study are indicated in blue. Boostrap values above 70% are indicated as black circles on their respective nodes.

Aside from the retrieved honey bee virus sequences, three near-complete genomes of *Iflaviridae*-like sequences were retrieved that were similar to *Drosophila*-infecting viruses, i.e., ThV, KiV and LJV. Phylogenetic reconstruction revealed these viruses clustering closely together with their nearest BLASTn hits (ThV, 95% nucleotide similarity with KP714072.1, KiV, 83% nucleotide similarity with KP14071.1 and LJV, 90% nucleotide similarity with KP14074.1) (Figure 6). The high similarity with *Drosophila*-infecting viruses, combined with the fact that the retrieved sequences can be assigned to the same viral species as their closest BLASTn hits (under the assumption that *Iflaviridae*-like species can be demarcated with a 90% amino acid cut-off of the putative capsid protein [64]) could imply that these viruses can replicate in both Diptera and Hymenoptera.

### 3.6. LJV and KiV Transmission and Replication

In order to understand the routes of transmission of both LJV and KiV, we investigated their presence in varroa mites and in the pupal stage of the honey bee. RT-qPCR analysis confirmed the presence of these two viruses in six out of 10 varroa mite samples (Ethi_1, Ethi_2, Ethi_3, Ethi_4, Ethi_7, and Ethi_10). However, LJV and KiV were not found in any of the pupa samples (infested by varroa or not).

**Figure 7:**
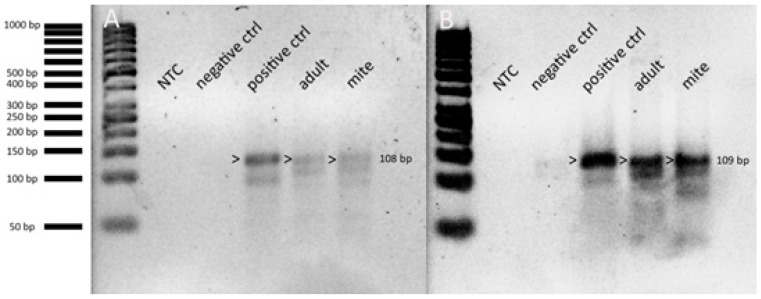
Inverted image of the high-resolution analysis of Reverse Transcriptase Multiplex Ligation Probe Amplification (RT-MLPA) to detect the negative strand intermediate of LJV in (**A**) and KiV in (**B**) in samples of adult bees and varroa mites. RT-MLPA analysis was performed using 4% high-resolution gel electrophoresis with a GeneRuler 50 bp ladder (Thermo Scientific). For each virus, the synthetic negative strand was used as positive control. We included also a non-infected honey bee adult as a negative control and a no-template control (NTC). Bands marked with an arrowhead confirm the presence of negative strand intermediate.

Then, we determined whether both viruses were actively propagating in the two samples types where they were found (i.e., adult honey bees and varroa mites). This was done by the detection of their negative strand, the intermediate of viral replication, using the RT-MLPA technique. RT-PCR has been used this purpose in the past, but it was proven to be sensitive to the miss-priming and self-priming of RNA during reverse transcription [65]. On the contrary, RT-MLPA is less prone to such false positive results, making it the preferred technique to detect the negative strand RNA intermediate. Instead of amplifying the original target, RT-MLPA will amplify a probe in a strand-specific manner through the ligation of two oligonucleotide half-probes hybridizing to a complementary cDNA target. This NAD-dependent ligase-65 is unable to ligate RNA–DNA hybrids, thus effectively ruling out false positive results. The negative strands of both viruses were found in both adult honey bees and varroa mites (Figure 7). However, we cannot exclude that the negative strand detection in varroa mites is the result of ingested honey bee cells [15].

## 4. Discussion

The occurrence of the *V. destructor* in Ethiopia was confirmed for the first time in a survey of 2010 that was conducted after the first reports of colony losses [66]. Varroa mites were found in all sampled districts of the Tigray region, and the mite infestation coincided with discolored and shrunken pupae [66]. In a follow-up study, honey bee colonies were examined on a monthly basis for three consecutive years (2011–2013), demonstrating the seasonal dynamics of mite infestation, but more importantly, that the effect of parasitosis on colony development was minor [67]. It seemed that in an extremely short time, the Ethiopian honey bees managed to cope with mite infestation. In a previous study, we examined the factors that restrain the population growth of *V. destructor* in Ethiopian honey bees (*A. m. simensis*) [68]. Here, we focused on the virus infections.

The present study explored the diversity of viruses in Ethiopian honey bees using an unbiased metagenomics approach combined with the NetoVIR enrichment protocol. We could identify five well-known honey bee viruses and 25 atypical viruses, most of which were never found in *A. mellifera* before. These viruses belong to *Dicistroviridae, Iflaviridae, Partitiviridae, Parvoviridae, Potyviridae, Secoviridae*, *Tombusviridae,* and taxonomically unclassified families (Tables S1–S3). It appeared that the addition of an enrichment step to the metagenomics protocol resulted in the identification of a wealth of virus. Often, the relatively low abundance of viral genomes makes virus diversity studies rather challenging [43,69]. In the present study, we increased the proportion of the reads of virus origin up to 25.6 million or 43.3%, compared to 28.5 million or 48.2% for the Eukaryota. This highlights that most of the host genome and transcriptome was removed during the NetoVIR enrichment protocol. The majority of the viral sequences detected in our study belonged to the *Iflaviridae* (Figure 2).

The known honey bee viruses identified include DWV, SBV, BQCV, AmFV, and LSV. In the metagenomics study, AmFV, which is a DNA virus [70], was found in one out of 10 sampling sites. The AmFV genome consists of 496.4 kb nucleotides (GenBank: KR819915.1). The identified AmFV scaffold has a length of 5813-nt. It showed 79.9% amino acid identity with AmFV isolated from honey bees in Switzerland (GenBank: YP_009165988.1). The presence of AmFV in honey bees was also confirmed previously in South Africa [29,31,71], Algeria [72], and northern Africa [73]. Data of the occurrence of this virus in other African countries is often lacking, simply because the monitoring programs focus on RNA viruses alone [26].

DWV is generally considered a serious threat of honey bee health and has been found to be strongly associated with varroa mite infestation [13,14,18]. In the present study, it was found in all monitored honey bee colonies (Figure 4). Overall, the DWV read counts represented 71.43% of the total viral reads. The DWV genomic sequences obtained in the present study fell into a clade with other type B variants of the DWV. DWV-B was previously designated as Varroa destructor virus-1, which was a virus originally isolated from *V. destructor* [74], but it has since been reported to replicate in honey bees, where it has been shown to cause clinical signs (wing deformities) [75]. Two other DWV types have been described so far: DWV-A is usually associated with symptomatic DWV in the presence of varroa [17], and DWV-C has more recently been found in Devon, England [76]. The reduction in DWV variant diversity that we found in the present study is typical for varroa-dependent transmission and was first described when the previously varroa-free Hawaiian island became invaded by the mite [17]. However, the individual effects of the different DWV genotypes on honey bee health is complex [77]. In continental Europe, DWV-B is associated with elevated virulence [78], while in the USA, England, and Wales, it was routinely detected in high virus loads in surviving colonies [79]. In the same study, DWV-A has been associated with higher overwintering colony losses [79]. Thus, our finding of only DWV-B fits well in the process of dominance of a certain strain in a geographic region. Given the apparently harmless infestation by the varroa mite of Ethiopian bees [68], DWV-B most probably has lower virulence in this part of Africa. However, we found a relatively large diversity of DWV-B in the Ethiopian strains, when compared to the relevant reference sequences used in our phylogenetic analysis. A similar divergence from the known reference sequences was observed for LSV, BQCV, and SBV. Further investigation is needed to depict whether this is a geographic region-dependent phenomenon.

Quantification of the virus burden per bee demonstrated that virus levels in Ethiopian honey bees are of the same magnitude as their European counterparts [80,81]. On the other hand, clinical symptoms of viral diseases are extremely rare in Ethiopia, which is confirmed in the present study. Honey bees in Ethiopia are apparently unable to suppress virus replication, but they may prevent its adverse effects, which is a phenomenon that was also observed in Gotland bees [82,83] and that is best described as virus tolerance [84]. In both cases, the bee populations survived without mite control measures, and therefore, it seems reasonable to believe that the submission to natural selection is likewise the basis of the increased resilience of Ethiopian honey bees. A possible mechanism of virus tolerance is mentioned here above, in particular, the enrichment of the less dangerous DWV B-type. Of course, this does not explain the total lack of clinical symptoms of for instance sacbrood virus, which was nevertheless found in sufficient numbers. We will further focus on this in a follow-up study.

We have also identified 15 viruses that were homologous to plant-specific viruses and belong to the families *Partitiviridae*, *Secoviridae*, *Tombusviridae*, and *Potyviridae*, which primarily infect plants [85,86]. Previous studies on the viral diversity in honey bees also detected viruses that primarily infect plants [31,38,72,87,88]. *Partitiviridae* and *Secoviridae* families have been described recently in honey bee samples from different countries across different continents [31,38,42,87]. The detection of these viruses in the present study may suggest a broad global distribution. The presence of sequences related to plant viruses in bee samples most likely represents environmental contamination and does not necessarily mean that these viruses are infectious to bees and may cause related health problems [38]. The role that bees play as vector of plant diseases caused by viruses is well described [89]. Cryptic viruses such as Redclover cryptic virus (ReCV1) and Vicia cryptic virus (VCV), which are members of *Partitiviridae*, are transmitted through pollen [90,91,92,93,94]. Bees can carry such plant viruses from infected pollen particles that are attached to their body [38] or that end up in their alimentary tract after ingestion. Virus-contaminated pollen can cause inter-taxa transmission of viruses and potentially impact non-*Apis* Hymenopteran species [95]. It has been reported that a plant virus, i.e., the Tabacco ringspot virus (TRSV)—a member of the *Secoviridae* family—replicates in bees and was found to have a negative effect on colony survival [87], but these findings were later disputed (http://www.microbe.tv/twiv/twiv-271-to-bee-or-not-to-bee-that-is-the-infection/). Nevertheless, it is noteworthy to study the impact of these plant-specific viruses associated with bees.

In addition, three viruses that previously were identified in *Drosophila* spp. were discovered in this study: LJV, KiV, and ThV [63,96,97]. Sequences related to ThV and LJV have recently also been detected in Australian honey bees [33]. To the best of our knowledge, this is the first report describing these two viruses in African bees. It may be indicative of a broader distribution and association with *A. mellifera*. Remarkably, our study suggests that LJV and KiV replicate in both the varroa mite and adult bees, but not in pupae. This would implicate a function of the mite as a virus reservoir as seen elsewhere [72], and that the vectoring of these viral agents occurs primarily through the phoretic stage of the mite. Virus infection of adult bees through virus-transmitting phoretic mites is only one of the horizontal transmission routes described for DWV [14]. Further research is needed to fully unravel the role of the mite in the transmission of these viruses to honey bees and their impact on the health of honey bees and mites. Moreover, our phylogenetic analysis revealed high similarity with *Drosophila*-infecting viruses and possibly denotes an unexpected inter-taxa transmission of viruses between Diptera and Hymenoptera.

DsDNV is a DNA virus that belongs to *Parvoviridae* family [98] and is an insect-specific virus. It causes high mortality in its natural host, the sugarcane borer *Diatraea saccharalis*. Here, the sequences related to DsDNV were found in two sampling sites with high read counts (74,733 reads). The presence of DsDNV-like virus with high read count and its virulence in its natural host may warrant further investigation on the impact of the virus on *A. mellifera*. Another densovirus was recently found in *Bombus terrestris* and *Bombus cryptarum* [31,42]. Finally, two more insect viruses that primarily infect mosquito spp. were found: Wenzhou sobemo-like virus 4 and Hubei mosquito virus 2, both with a low distribution across the samples (one locality).

Nationwide health monitoring programs of honey bees often provide information of the prevalence of viruses and their importance in the context of bee health [99,100,101]. However, such studies mostly focus on a small set of known bee viruses and give no insight into the so-far undiscovered viral species to which bees are exposed. Metagenomic studies such as the present study expand our knowledge of the bee virome, but they fail to answer questions about their distribution on a larger scale, virulence, and impact on bee health. Therefore, local monitoring programs should expand their scope and focus also on the atypical viruses that we discovered. In order to make this feasible, multiplex virus fingerprinting techniques are needed that allow—in a single run—to collect data on the presence of a much broader set of viruses. In 2012, De Smet et al. [62] launched the use of the Multiplex Ligation-dependent Probe Amplification (MLPA) technique in the context of bee virus detection. The test was called BeeDoctor, and it enables detecting 10 bee viruses in a single run. However, theoretically, the MLPA technique allows increasing the number of target nucleic acid sequences up to 40 [102]. With the wealth of new viruses found to be associated with bees, there is an urgent need for an updated version of the BeeDoctor or similar multiplex techniques. This would enable us to explore the importance of this richness and diversity of bee viruses.

## Supplementary Materials

The following are available online at https://www.mdpi.com/1999-4915/12/11/1218/s1, Figure S1: Bioinformatics workflow, Figure S2: Phylogenies of BQCV and SBV, Table S1: Primers and probes used for RT-qPCR and negative strand MLPA, Table S2: Identified known honey bee viruses, Table S3: Identified atypical viruses that seem plant-specific, Table S4: Identified atypical viruses that seem insect-specific. Multifasta-file of the sequences retrieved in this study and used in the phylogenies of the known honey bee viruses.
