# Supplementary material for: Metagenomic Approach with the NetoVIR Enrichment Protocol Reveals Virus Diversity within Ethiopian Honey Bees (Apis mellifera simensis)
Source: Viruses. 2020 Oct 27;12(11):1218. doi: 10.3390/v12111218 (PMC7692050; doi:10.3390/v12111218)
Supplement: Supplementary file 1 [file viruses-12-01218-s001.pdf]

Figure S1. Bioinformatics workflow. Workflow used for the discovery of taxon from the next generation sequencing dataset from the Illumina NextSeq**.**

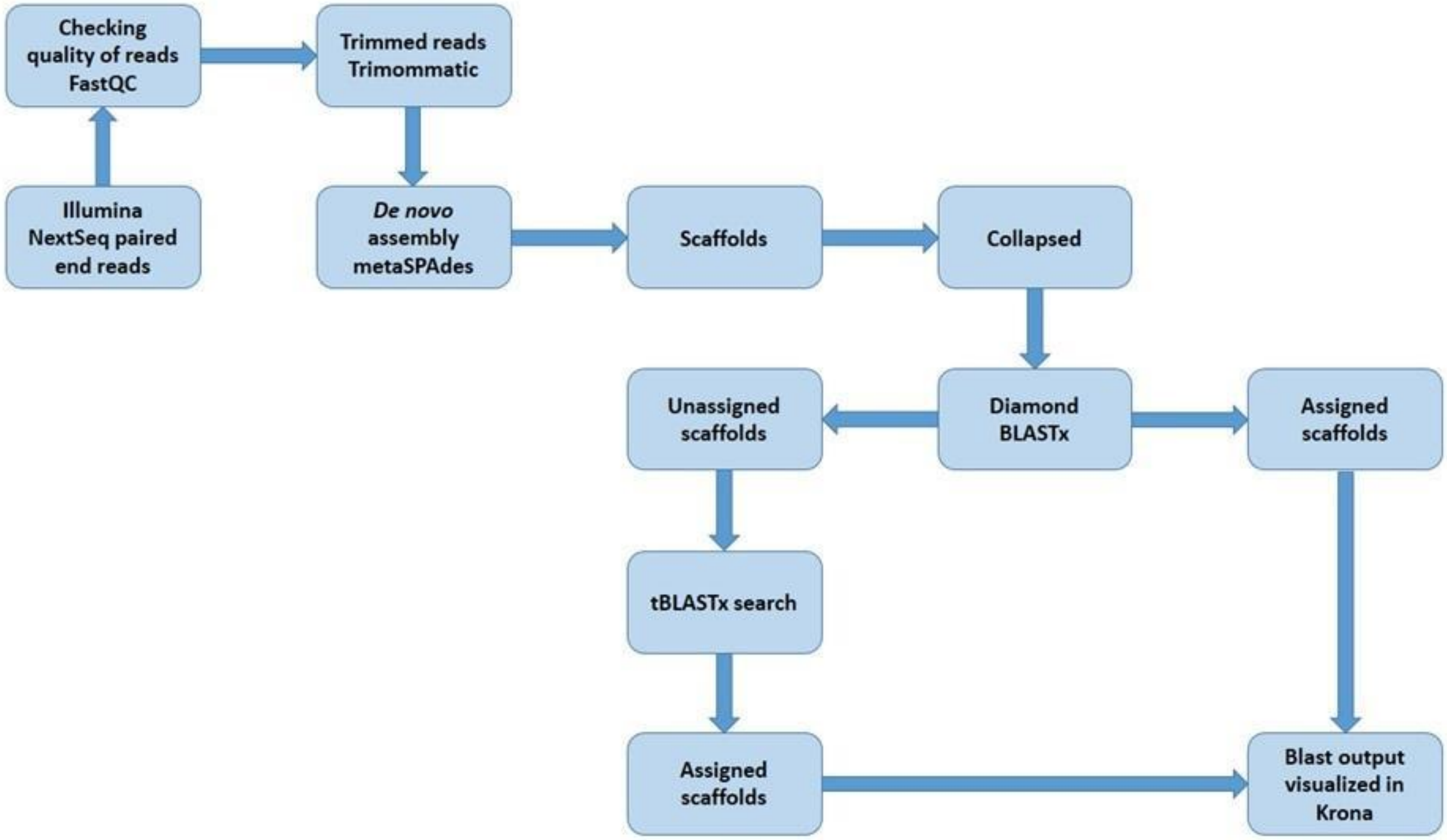

Figure S2. Phylogenies of BQCV and SBV. Outgroup-rooted phylogenetic trees were drawn based on non-structural protein (BQCV) or polyprotein (SBV) alignments (on amino acid level). Bootstrap values exceeding 70% are indicated as black circles on their respective nodes. Tip labels as well as branch groups are colored according to the geographical location of which the sequence was derived. Sequences retrieved in this study are indicated in blue.

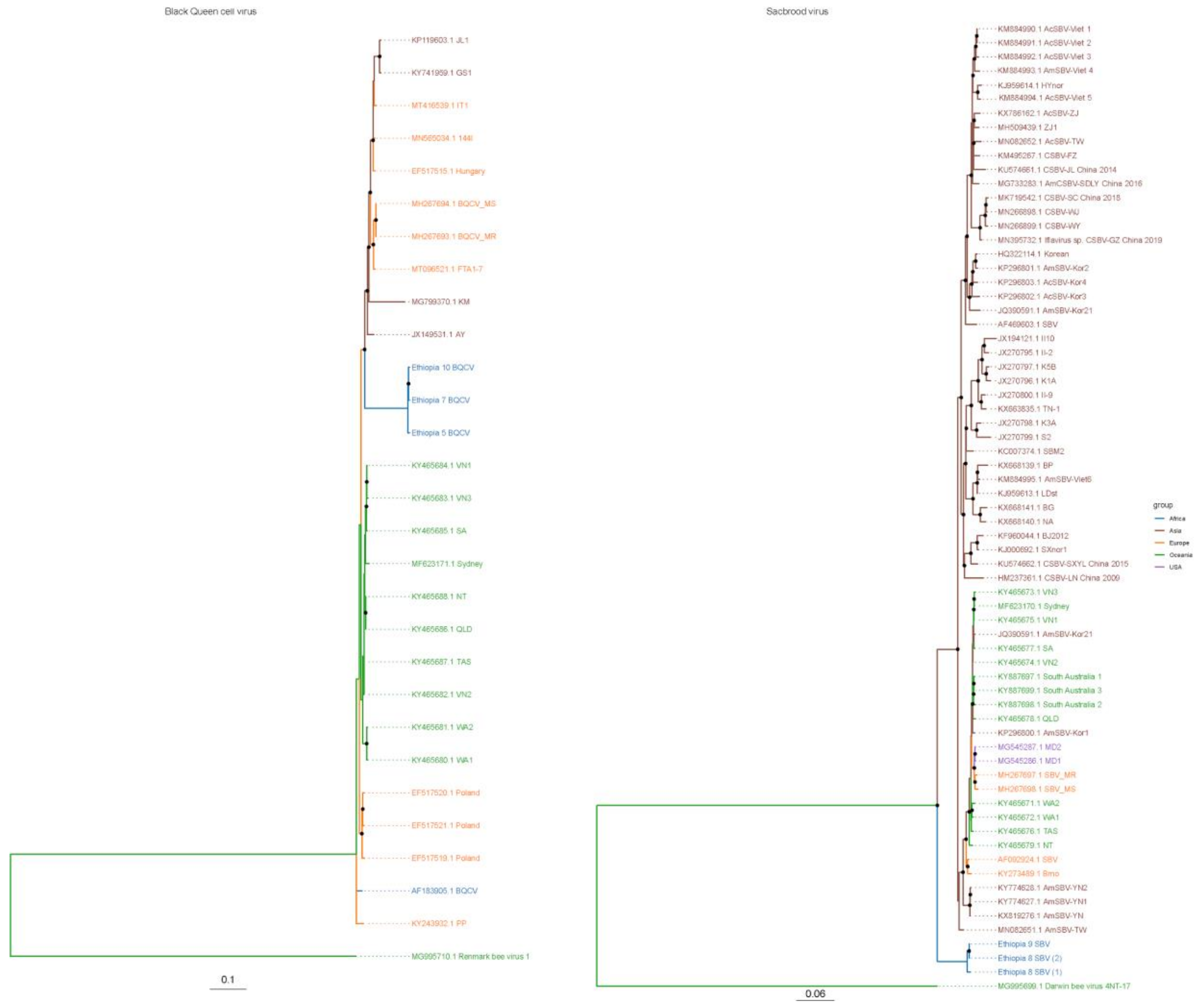

Table S1. Primers and probes used for RT-qPCR and negative strand MLPA. LPO: left probe oligo; RPO: right probe oligo; F: forward primer; R: reverse primer; Size: size of the amplicon; Target sequences of the MLPA probes are underlined; KiV: Kilifi virus; LJV: La Jolla virus; DWV: Deformed wing virus; BQCV: Black queen cell virus; SBV: Sacbrood virus.

**Virus**	**Strand**	**Function**	**Sequence (5'-3')**	**Size (bp)**	**References**
KiV	neg	(-) cDNA	TTGGGATCAGGTCAAAGTCC	109	This study
		(-) MLPA-LPO	GGGTTCCCTAAGGGTTGGAGAAGGCCATAGATGTTGGAGTGACAGCATTGTGT		This study
		(-) MLPA-RPO	$^{P-}$ACAGCTACCACCTTAAGGCCTTGGTTAGTACGATCTAGATTGGATCTTGCTGGCAC		
	pos	RT-qPCR F	ACGGAGGAAATGCTGGTAGTTT	130	This study
		RT-qPCR R	CATACAAGGTTTAGGGTCGGCATC		
LJV	neg	(-) cDNA	CCGACTTTGAAGCCAAGAAA	108	This study
		(-) MLPA-LPO	GGGTTCCCTAAGGGTTGGATGGAATACCGCAAAAACACGTCGCTACCGTTAT		This study
		(-) MLPA-RPO	$^{P-}$AACCGAACTTACCGCACGTTTGACATTGGAGGTTCTAGATTGGATCTTGCTGGCAC		
	pos	RT-qPCR F	TATTCTGGAGTGCTGGTGATGATTT	113	This study
		RT-qPCR R	CATTTGCGTGGAGTGATTG		
DWV	pos	RT-qPCR F	CGTCGGCCTATCAAAG	413	[52]
		RT-qPCR R	CTTTTCTAATTCAACTTCACC		
BQCV	pos	RT-qPCR F	AGTGGCGGAGATGTATGC	294	[53]
		RT-qPCR R	GGAGGTGAAGTGGCTATATC		
SBV	pos	RT-qPCR F	TTGGAACTACGCATTCTCTG	335	[53]
		RT-qPCR R	GCTCTAACCTCGCATCAAC		

Table S2. Identified known honey bee viruses. Total number of reads assigned to the longest scaffold, accession IDs of the best BLASTx hit, sequence (AA) identities (%) and the length of the longest scaffold (nt) are given.

**Most closely related known virus**	Genome type	Family	Sample code	Total reads	Best BLASTx hit	AA identity (%)	length (nt)
Deformed wing virus (DWV)	ssRNA	*Iflaviridae*	Ethi_1, Ethi2, Ethi_3, Ethi_5, Ethi_6, Ethi_7, Ethi_8, Ethi_9, Ethi_10	18,309,545	QHR77116.1	98.34	10,986
Black queen cell virus (BQCV)	ssRNA	*Dicistroviridae*	Ethi_1, Ethi_2, Ethi_3, Ethi_5, Ethi_6, Ethi_7, Ethi_10	1,554,072	QKW94190.1	89.03	8,504
Lake Sinai virus 1 (LSV-1)	ssRNA	Unclassified Sinaivirus	Ethi_3, Ethi_5, Ethi_7	1,278,162	YP_009388494.1	76.93	5,731
Sacbrood virus (SBV)	ssRNA	*Iflaviridae*	Ethi_5, Ethi_8, Ethi_9	377,748	NP_049374.1	92.41	8,770
Lake Sinai virus NE (LSV-NE)	ssRNA	Unclassified *Sinaivirus*	Ethi_3, Ethi_5, Ethi_7	264,012	YP_009388494.1	81	5,731
Apis mellifera filamentous virus (AmFV)	dsDNA	Unclassified dsDNA viruses	Ethi_1	2,310	YP_009165988.1	80	5,813

Table S3. Identified atypical viruses that shared homology with plant-specific viral sequences. Total number of reads assigned to the longest scaffold, accession IDs of the best BLASTx hit, sequence (AA) identities (%) and the length of the longest scaffold (nt) are given.

**Novel virus**	Genome type	Family	Sample code	Total Reads	Best BLASTx hit	AA identity (%)	length (nt)
Red clover cryptic virus 1 (ReCV1)	dsRNA	*Partitiviridae*	Ethi_1, Ethi_3, Ethi_5, Ethi_7, Ethi_9	45,819	AWK57378.1	82.79	1,978
Melon mild mottle virus	ssRNA	*Secoviridae*	Ethi_1	22,448	AWK77905.1	67.89	7,155
Dill cryptic virus 2 (DCC-2)	dsRNA	*Partitiviridae*	Ethi_3, Ethi_5	4603	YP_007891054.1	99.73	2,385
Spinach cryptic virus 1 (SPCV 1)	dsRNA	*Partitiviridae*	Ethi_1, Ethi_5	18,159	YP_009508045.1	58.74	1,733
Olive latent ringspot virus (OLRV)	ssRNA	*Secoviridae*	Ethi_1	15,361	YP_009508093.1	32.13	3,526
Crimson clover cryptic virus 2 (CCCV-2)	dsRNA	*Partitiviridae*	Ethi_5	6,425	YP_009508060.1	76.11	2,267
Dill cryptic virus 1 (DCV1)	dsRNA	*Partitiviridae*	Ethi_3	10,820	YP_008719880.1	99.03	1,942
Bipolaris maydis partitivirus 1 (BmPV1)	dsRNA	*Partitiviridae*	Ethi_3	11,454	QGW36011.1	61.28	1,793
Grapevine Anatolian ringspot virus (GARSV)	ssRNA	*Secoviridae*	Ethi_1	3,705	YP_006576511.1	80.28	4,181
Pea seed-borne mosaic virus (PSbMV)	ssRNA	*Potyviridae*	Ethi_5	2,444	CAB85904.1	95.90	7,072
Vicia cryptic virus (TVC)	dsRNA	*Partitiviridae*	Ethi_1	3,621	ABN71237.1	77.68	766
Turnip crinkle virus (TCV)	ssRNA	*Tombusviridae*	Ethi_1	2,485	P17460.3	94.08	3,597
Rose partitivirus (RoPV)	dsRNA	*Partitiviridae*	Ethi_3	2,316	YP_009551597.1	79.68	1,830
Rose cryptic virus 1 (RoCV1)	dsRNA	*Partitiviridae*	Ethi_5	1,204	ABV89762.1	81.30	1,721
Hop trefoil cryptic virus 2 (HTCV2)	dsRNA	*Partitiviridae*	Ethi_1, Ethi_5	9,306	YP_007889825.1	77.75	2,363

Table S4. Identified atypical viruses that shared homology with insect-specific viral sequences. Total number of reads assigned to the longest scaffold, accession IDs of the best BLASTx hit, sequence (AA) identities (%) and the length of the longest scaffold (nt) are given.

**Novel virus**	Genome type	Family	Sample code	Total Reads	Best BLASTx hit	AA identity (%)	length (nt)
La Jolla virus (LJV)	ssRNA	*Iflaviridae*	Ethi_1, Ethi_10	11,928	YP_009140562	96.60	10,262
Kilifi virus (KiV)	ssRNA	unclassified *Picornavirales*	Ethi_1, Ethi_10	4,829	AYQ66683	85.20	8,963
Thika virus (ThV)	unclassified RNA viruses	Unassigned virus families	Ethi_1	2,259	YP_009140561.1	97.26	6,169
Diatraea saccharalis densovirus (DsDNV)	ssDNA	*Parvoviridae*	Ethi_2, Ethi_5	74,733	NP_046815.1	46.07	503
Wenzhou sobemo-like virus 4	RNA	Unclassified	Ethi_5	2,678	QEM39285.1	75.26	2,549
Hubei mosquito virus 2	RNA	Unclassified	Ethi_5	10,005	YP_009259484.2	47.17	1,517
Hubei toti-like virus 2	RNA	Unclassified	Ethi_5	40,367	YP_009336496.1	62.38	5,013
Hubei partiti-like virus 51	RNA	Unclassified	Ethi_4, Ethi_5	2,959,222	APG78321.1	77.05	1,049
Hubei partiti-like virus 34	RNA	Unclassified	Ethi_4, Ethi_5	315,396	APG78322.1	95.20	1,458
Wuhan insect virus 27	RNA	Unclassified	Ethi_7, Ethi_8	12,782	YP_009342434.1	32.87	4,765
